# Missed and Delayed Diagnoses of Acute Human Immunodeficiency Virus (HIV) Infection in a Southern Opt-Out HIV Testing Environment Without Reflex HIV RNA Testing

**DOI:** 10.1093/ofid/ofae684

**Published:** 2025-01-17

**Authors:** Sarah F Gruber, Eli P Wilber, Brittany Smith, Paulina A Rebolledo, Jonathan A Colasanti, Rishika Iytha, Megan Schwinne, Chad Robichaux, Meredith H Lora

**Affiliations:** Department of Medicine, Emory University School of Medicine, Atlanta, Georgia, USA; Division of Infectious Diseases, Department of Medicine, Emory University School of Medicine, Atlanta, Georgia, USA; Grady Health System, Atlanta, Georgia, USA; Grady Health System, Atlanta, Georgia, USA; Division of Infectious Diseases, Department of Medicine, Emory University School of Medicine, Atlanta, Georgia, USA; The Ponce Center, Grady Health System, Atlanta, Georgia, USA; Rollins School of Public Health, Emory University, Atlanta, Georgia, USA; Division of Infectious Diseases, Department of Medicine, Emory University School of Medicine, Atlanta, Georgia, USA; Rollins School of Public Health, Emory University, Atlanta, Georgia, USA; Department of Biomedical Engineering, Georgia Institute of Technology, Atlanta, Georgia, USA; Department of Biomedical Informatics, Emory University School of Medicine, Atlanta, Georgia, USA; Department of Biomedical Informatics, Emory University School of Medicine, Atlanta, Georgia, USA; Department of Medicine, Emory University School of Medicine, Atlanta, Georgia, USA; Division of General Internal Medicine, Department of Medicine, Emory University School of Medicine, Atlanta, Georgia, USA

**Keywords:** acute HIV infection, discordant results, HIV diagnostic delay, HIV testing, reflex HIV RNA

## Abstract

Prompt confirmation of human immunodeficiency virus (HIV) is critical. We examined 10 years of discordant results without reflex HIV RNA. Of patients with acute HIV infection, 43.9% (95% confidence interval, 36.2%–52.0%) had confirmation delays >30 days or were never confirmed, indicating a need for reflex RNA to facilitate diagnosis.

## Teaser

Prompt confirmation of HIV is critical. We examined ten years of discordant results without reflex HIV RNA. 43.9% of patients with acute HIV infection had confirmation delays exceeding 30 days or were never confirmed, indicating a need for reflex RNA to facilitate diagnosis.

An estimated 13% of the 1.2 million people with human immunodeficiency virus (HIV; PWH) in the United States are unaware of their status [1, 2]. Early diagnosis of HIV is a primary pillar of the national Ending the HIV Epidemic plan in the United States [3]. Sexual transmission during acute HIV infection (AHI) is up to 26 times higher than during chronic infection, and, despite its short duration, AHI accounts for up to 50% of all new HIV-1 transmissions [4–8]. Diagnosis and treatment during AHI lead to improved outcomes for PWH [9–12] and are crucial to limit the spread of HIV [6–8].

To increase HIV screening rates and facilitate prompt diagnosis of HIV infection, the US Centers for Disease Control and Prevention (CDC) recommends one-time opt-out HIV testing for all patients aged 13–64 years and annual screening for persons at increased risk of HIV [13–17]. CDC HIV testing guidelines recommend an HIV antigen/antibody screening assay, followed by an HIV-1/HIV-2 antibody differentiation immunoassay if screening results are reactive [18, 19]. If the differentiation immunoassay result is negative or indeterminate (discordant test result), an HIV RNA nucleic acid test (NAT) is needed to distinguish between AHI and false-positive (FP) findings [18, 19]. If NAT is not performed on the original blood sample (ie, reflex NAT), a second blood sample is required; complexity and cost limit widespread implantation of reflex NAT [18, 19].

Our urban safety net healthcare system in Atlanta, Georgia implemented opt-out HIV testing in 2013, with a testing algorithm that aligns with CDC guidelines but does not include reflex NAT for discordant results. The current study examines discordant HIV test results from an opt-out HIV testing environment over a 10-year period. We report AHI diagnoses, unconfirmed results, and time to confirmation of HIV status for patients with discordant HIV testing.

## METHODS

This retrospective cohort study was conducted at Grady Health System (GHS), an urban safety-net hospital in Atlanta, Georgia. In 2013, GHS implemented opt-out HIV testing (following CDC guidelines; see Supplementary Figure 1) and an HIV navigator to provide disclosure and linkage support for people with positive HIV test results. For nonhospitalized patients with discordant results in whom confirmatory RNA has not been performed, the patient navigator coordinates follow-up NAT via phone calls (ie, outreaches).

HIV testing data was collected from adult patients (aged ≥18 years) with ≥1 discordant HIV test result between 1 January 2012 and 31 December 2022. A discordant test result was defined as a reactive screening assay result followed by a negative or indeterminate antibody differentiation assay result (2015 onward) or Western blot (before 2015). Age, race, ethnicity, and sex assigned at birth were recorded. The date of the initial positive discordant test result, the date of repeated testing, hospitalized status at the time of the discordant result, and the number of testing-related patient outreaches between initial discordant result and confirmation of status were abstracted.

Laboratory and prescription data, digitally accessible outside records, and clinical notes were manually reviewed to determine HIV status, which was classified as AHI, FP, or unknown. AHI was defined as a discordant screen followed by a positive viral load within 3 months or a discordant screen without confirmation testing but accompanied by signs or symptoms concerning for AHI in a patient with risk factors. In cases where the AHI diagnosis was unclear, a second clinician independently reviewed the data to determine HIV status. FP was defined as a discordant screen with subsequent negative screening results, a negative viral load within 3 months, or multiple discordant tests over time. All other patients were classified as unknown.

This research was approved by the Emory University Institutional Review Board and the Grady Health Systems Research Oversight Committee with a waiver for informed consent. No patient images or otherwise patient-identifying information are included in this report.

We determined the proportion of AHI, FP, and unknown cases and compared rates by patient age, race, ethnicity, and sex assigned at birth. We examined the time to confirmation for patients who were not hospitalized at the time of discordant testing. We determined the median time to confirmation and interquartile range (IQR) for the total cohort and by HIV status. Using RStudio software version 2024.04.2 +746 [20], we determined 95% confidence intervals (CI) for the proportion of the cohort remaining unconfirmed 7, 14, 30, and 365 days after the initial discordant result, as well as those without confirmatory testing. We calculated the mean and median number of patient outreaches, excluding those who were hospitalized at the time of discordant testing or whose confirmatory testing was sent on the same day as the discordant result. If no outreach calls were documented for a given patient, they were considered to have 0 outreaches. Figures were created using r package “ggplot2.”

## RESULTS

Among 88 524 unique patients who received HIV testing at GHS during our study period, 585 had ≥1 discordant result. The sociodemographic characteristics of the cohort are displayed in Table 1; 195 patients (33.5%) were classified as AHI, 301 (51.5%) as FP, and 88 (15.0%) as unknown (Table 1).

**Table 1.** Demographics and Time to Confirmation by Human Immunodeficiency Virus Status

Variable	Patients, No. (% of Total)			
	AHI (n = 195 [33.5%])	FP (n = 301 [51.5%])	Unknown (n = 88 [15.0%])	Total (n = 585)^a^
Race				
Hispanic	7 (1.2)	30 (5.1)	5 (0.5)	40 (6.8)
Non-Hispanic black	175 (29.9)	238 (40.7)	68 (11.6)	481 (82.2)
Non-Hispanic white	10 (1.7)	21 (3.6)	6 (1.0)	37 (6.3)
Other	4 (0.7)	12 (2.1)	11 (1.9)	27 (4.6)
Sex assigned at birth				
Female	45 (7.7)	172 (29.4)	34 (5.8)	251 (42.9)
Male	151 (25.8)	129 (22.1)	54 (9.2)	334 (57.1)
Age at discordant result, y				
<25	54 (9.2)	47 (8.0)	14 (2.4)	115 (19.7)
25–35	80 (13.7)	66 (11.3)	21 (3.6)	167 (28.6)
35–44	23 (3.9)	44 (7.5)	22 (3.8)	89 (15.2)
>45	39 (6.7)	144 (24.6)	31 (5.3)	214 (26.6)
Nonhospitalized patients, no. remaining unconfirmed (column %)	148 (29.1)	273 (53.6%)	88 (17.3%)	509^b^
Time from initial discordant result, d				
7	93 (62.8)	189 (69.2)	88 (100.0)	370 (72.7)
14	72 (48.7)	149 (54.6)	88 (100.0)	309 (60.7)
30	65 (43.5)	130 (47.6)	88 (100.0)	283 (55.6)
365	43 (29.1)	52 (19.1)	88 (100.0)	183 (36.0)
Never confirmed	33 (22.3)	0 (0)	88 (100.0)	121 (23.8)

Abbreviations: AHI, acute human immunodeficiency virus (HIV) infection; FP, false-positive HIV screening assay; IQR, interquartile range.

^a^Total number of patients with discordant HIV test results.

^b^Total number of patients with discordant HIV test results, excluding those who were hospitalized at the time of discordant testing or admitted during the same encounter.

**Figure 1.**
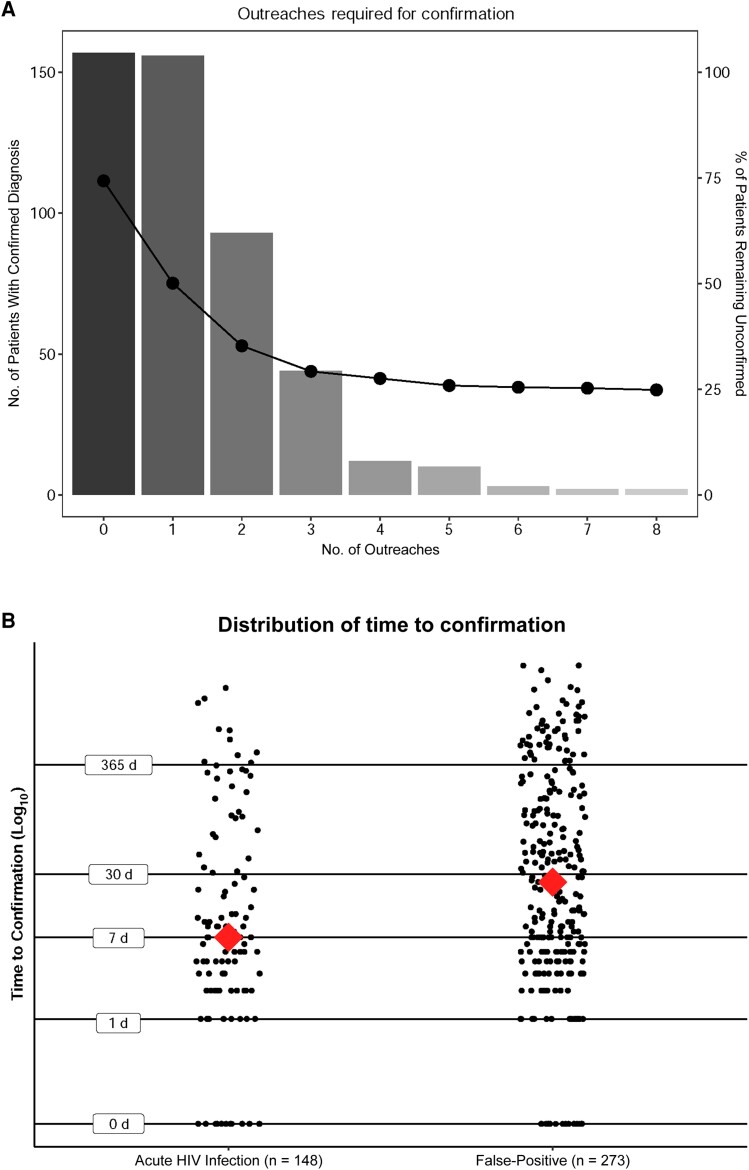
*A*. Number of patients receiving a confirmed diagnosis per number of outreach attempts. Hospitalized patients and those with same-day confirmatory testing are excluded. Line shows the percentage of the cohort remaining without a diagnosis after each number of outreach attempts. *B*. Distribution of time to confirmation (log-transformed) for the acute human immunodeficiency virus (HIV) infection and false-positive groups. Diamonds represent group medians.

Of 509 patients with discordant testing results who were not hospitalized, 148 (29.1%) were classified as AHI, 273 (53.6%) as FP, and 88 (17.3%) as unknown (Table 1). Among patients with confirmatory testing, the median duration to confirmation (IQR) was 13 (4–135) days. At 30 days after the index screen, 55.6% (95% CI, 51.3%–59.9%) of patients remained unconfirmed (Figure 1*A*); 23.8% (20.3%–27.7%) never received confirmatory testing. Among patients with AHI, the median duration to confirmation (IQR) was 6.5 (2–31.5) days. At 30 days after the index screen, 43.9% (95% CI, 36.2%–52.0%) of patients with AHI remained unconfirmed (Figure 1*A*); 22.3% (16.3%–29.7%) of patients had a clinical diagnosis of AHI but never received confirmatory testing.

In total, 660 total outreaches were performed, with a range of 0–8 outreaches per patient (Figure 1*B*). The median number of patient outreaches per discordant result was 1 (IQR, 0–2), and the mean number was 1.3 (SD, 1.4).

## DISCUSSION

In our opt-out HIV testing environment without reflex HIV RNA testing, a third of patients with discordant HIV tests had AHI. Nearly half of nonhospitalized patients with AHI experienced confirmation delays exceeding 30 days or never received confirmatory testing or disclosure of their status.

These findings have important implications for patient outcomes and public health. Diagnosis and treatment during AHI lead to improved short- and long-term outcomes for PWH, as early treatment reduces viral reservoir size and chronic inflammation, limits disease progression, and preserves immune function [9–12]. In addition, detection of AHI limits the spread of HIV; up to 50% of all new diagnoses of HIV may be transmitted during the acute phase [4–8]. More than 40% of patients with AHI did not receive confirmation of diagnosis within 30 days, resulting in substantial opportunity for ongoing transmission. In a Chicago-based prospective study, Kaperak et al [18] demonstrated that implementation of reflex NAT increased timely confirmation of AHI and decreased the median time to linkage from 12.7 to 2.6 days. Our data suggest that among populations with high burdens of AHI, implementing reflex NAT could improve health outcomes and reduce HIV transmission by facilitating earlier diagnosis and linkage to care.

While patient navigation supported confirmation of HIV status, it did not bridge the shortcomings of the testing environment. HIV testing without reflex NAT requires a patient to have correctly documented contact information and to return multiple times: first for NAT and subsequently for HIV care linkage. These diagnosis and linkage barriers disproportionately affect socially vulnerable populations who are at risk for HIV and have limited access to healthcare [21]. The broad range of observed outreach attempts may reflect the challenges navigators face in supporting patients without phones or accessible transportation. By decreasing the number of visits required to diagnose HIV, implementing reflex NAT would improve patient outcomes, particularly among vulnerable populations, and allow navigators to focus on linkage and retention for people with confirmed HIV [18, 22].

The current study, the largest examining delays in AHI diagnosis within the context of discordant testing, supports the findings reported by Kaperak et al [18] before implementation of reflex NAT at their institution. Our data, collected in an area with one of the highest incidences of HIV in the United States—including 4 of the 48 Ending the HIV Epidemic priority counties, where 50% of all new diagnoses occur—highlight the need for improved diagnostic algorithms to facilitate early identification and rapid linkage to care for PWH [23]. Some experts argue that HIV NAT should replace the confirmatory immunoassay as the second test in the HIV diagnostic algorithm, given its high specificity for HIV-1, although confirmation would still be required in the case of negative RNA to identify HIV controllers (PWH with undetectable viral loads) and patients with HIV-2 [24]. Our findings suggest that funded efforts to support broader integration of reflex HIV NAT in areas of high HIV prevalence could decrease time to diagnosis, improve linkage to care, and break the chain of ongoing transmission.

Limitations of this study include the fact that it was conducted in a single safety net health system serving a racially homogenous patient population with a high incidence and prevalence of HIV. Additional prospective studies are needed to quantify the impact of reflex NAT on diagnosis timelines, missed diagnoses, and allocation of personnel time in other populations, including in lower-prevalence settings.

In conclusion, opt-out HIV testing without reflex NAT for discordant results contributes to missed and delayed diagnoses of AHI. Coordinating follow-up NAT is complicated by socioeconomic barriers and does not effectively facilitate early diagnosis and linkage. Reflex NAT to improve rapid diagnosis of AHI is essential for reducing transmission of HIV, particularly in areas of high HIV prevalence and in populations with increased barriers to accessing healthcare.

## Supplementary Material

ofae684_Supplementary_Data

## Supplementary Data

Supplementary materials are available at *Open Forum Infectious Diseases* online. Consisting of data provided by the authors to benefit the reader, the posted materials are not copyedited and are the sole responsibility of the authors, so questions or comments should be addressed to the corresponding author.
