# Supplementary material for: Missed and Delayed Diagnoses of Acute Human Immunodeficiency Virus (HIV) Infection in a Southern Opt-Out HIV Testing Environment Without Reflex HIV RNA Testing
Source: Open Forum Infect Dis. 2025 Jan 17;12(1):ofae684. doi: 10.1093/ofid/ofae684 (PMC11739807; doi:10.1093/ofid/ofae684)
Supplement: ofae684_Supplementary_Data [file ofae684_supplementary_data.pdf]

Supplementary Figure 1. Grady Health System opt-out HIV testing workflow for discordant results 2012-2022.

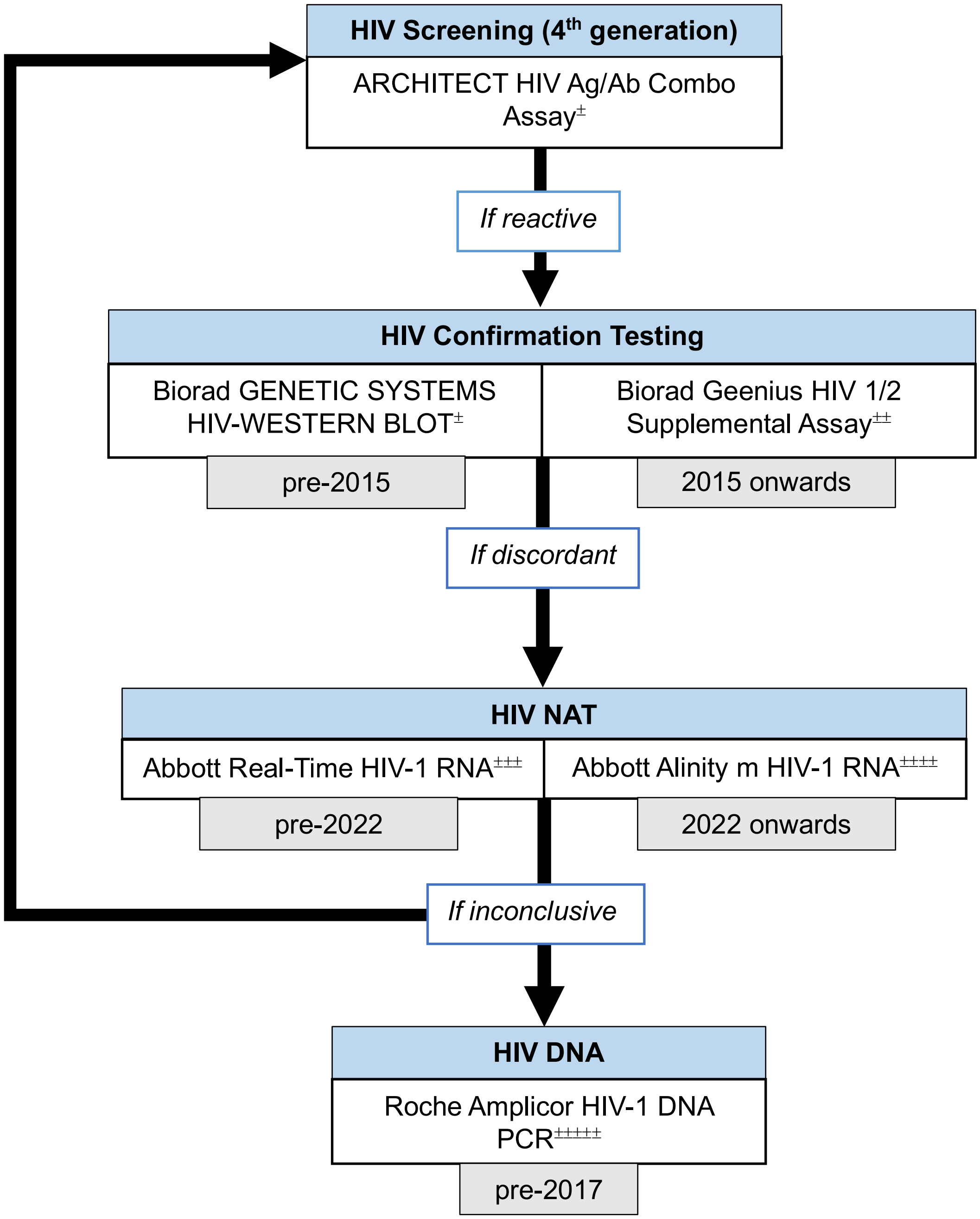

Footnote. HIV = Human Immunodeficiency Virus; Ab/Ab = Antigen/Antibody; Combo = combination; HIV 1/2 = HIV-1 and HIV-2; "discordant" = positive HIV screening and negative HIV confirmation testing; NAT = nucleic acid testing; ± requires serum or plasma specimens; ±± requires serum, plasma, or whole blood specimens; ±±± requires plasma or dried blood spot specimens; ±±±± requires serum, plasma, or dried blood spot specimens; ±±±±± requires whole blood specimens.
